# Capturing crop adaptation to abiotic stress using image-based technologies

**DOI:** 10.1098/rsob.210353

**Published:** 2022-06-22

**Authors:** Nadia Al-Tamimi, Patrick Langan, Villő Bernád, Jason Walsh, Eleni Mangina, Sónia Negrão

**Affiliations:** ^1^ School of Biology and Environmental Science, University College Dublin, Dublin, Ireland; ^2^ School of Computer Science and UCD Energy Institute, University College Dublin, Dublin, Ireland

**Keywords:** abiotic stress, imaging, high-throughput phenotyping, crops, machine learning

## Abstract

Farmers and breeders aim to improve crop responses to abiotic stresses and secure yield under adverse environmental conditions. To achieve this goal and select the most resilient genotypes, plant breeders and researchers rely on phenotyping to quantify crop responses to abiotic stress. Recent advances in imaging technologies allow researchers to collect physiological data non-destructively and throughout time, making it possible to dissect complex plant responses into quantifiable traits. The use of image-based technologies enables the quantification of crop responses to stress in both controlled environmental conditions and field trials. This paper summarizes phenotyping imaging technologies (RGB, multispectral and hyperspectral sensors, among others) that have been used to assess different abiotic stresses including salinity, drought and nitrogen deficiency, while discussing their advantages and drawbacks. We present a detailed review of traits involved in abiotic tolerance, which have been quantified by a range of imaging sensors under high-throughput phenotyping facilities or using unmanned aerial vehicles in the field. We also provide an up-to-date compilation of spectral tolerance indices and discuss the progress and challenges in machine learning, including supervised and unsupervised models as well as deep learning.

## Graphical abstract

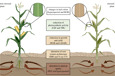

## 1. Introduction

Agriculture is facing tremendous challenges, resulting from the rapidly growing population, extreme weather events and serious loss of arable land and water resources. Crop yields are restricted inherently by plant stresses (biotic and abiotic), and plant breeders' efforts involve minimizing plant stress yield losses by incorporating and identifying resistance genes to develop more resilient varieties. Crops have optimum levels of nutrient and water availability and ideal temperature ranges for production. Environmental conditions outside of these ranges lead to abiotic stress and can interrupt normal plant physiology and ultimately lead to death. Abiotic stresses rarely occur in isolation and responses are highly variable depending on the crop, growth stage and combination of abiotic and biotic stresses found in the field [1]. Severe yield penalties have increased steadily in the past decades, and extreme weather events such as floods, drought and heat are predicted to increase as a direct consequence of climate change [2–4].

**Figure 1.**
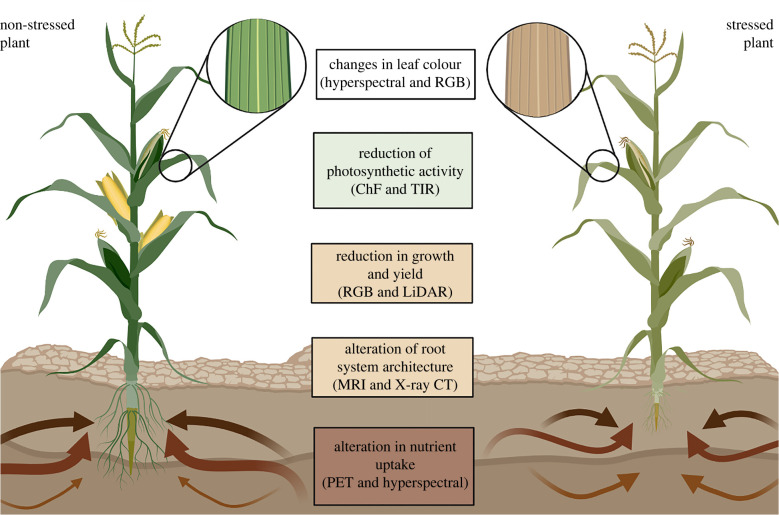
Summary of major crop physiological traits for screening abiotic stress responses, and imaging technologies to quantify them. The illustration on the left displays a plant under ideal conditions. On the right, predominant trait changes are observed under abiotic stress conditions. Maize is used as a hypothetical example, yet these physiological responses are common to other crops experiencing abiotic stress. Imaging technologies are listed in the centre. Abbreviations (from top to bottom): RGB, red, green and blue; ChF, chlorophyll fluorescence; TIR, thermal infrared imaging; LiDAR, light detection and ranging; MRI, magnetic resonance imaging; CT, computed tomography; PET, positron emission tomography.

Plant phenotyping plays a critical role in accurate and precise trait collection and use of genetic tools to improve plant performance. Phenotypes are determined from essentially all parts of plants ranging from the cellular level to the whole plant or canopy level [5] and can be defined as the application of methodologies to measure a specific plant trait. Image-based technologies can be used in phenotyping to identify plant changes in reflectance, biomass and thermal radiation (figure 1). The effects of and adaptations to abiotic stresses can be related to imaging data to target specific stress responses. Response traits can be identified when studying specific stressors, for example, low water availability or high temperatures. Understanding the underlying mechanisms of the stress under scrutiny, and the plant responses to it is critical to indicate which traits should be targeted in the phenotyping experiment. Stress responses have been documented in several overviews that are available for abiotic stresses such as drought [6], salinity [7], flooding [8], heat stress [9] and heavy metals [10].

Until recently, classical methods of phenotyping were not in the same league as the available high-throughput genome sequencing and genotyping methods. The past bottleneck in phenotyping has motivated scientists across different disciplines from agriculturists to engineers to integrate newer technologies in field phenotyping. Such advancements in phenotyping have led to an interdisciplinary research field that connects computer science, biology, remote sensing, statistics, and genomics with the aim of coupling complex plant traits to genetic expression, all for the need to achieve future food security. High-throughput phenotyping (HTP) has unraveled new possibilities for non-destructive phenotyping in plants for a large number of traits including abiotic stress traits (which is the main focus of the review) such as salinity, drought, flood, nutrient deficiency and other environmental stress factors. Plant-image phenotyping has been carefully reviewed by other authors (e.g. [11–17]); however, this review examines plant image-based technologies to dissect the complex physiology of abiotic stress responses. First, we discuss the different types of imaging systems, including their advantages and limitations to quantify abiotic stress and highlight the use of HTP. Next, we explore the use of imaging systems for abiotic stress studies and recognize their achievements in the dissection of tolerance mechanisms and breeding efforts. Finally, we explore the current advances in monitoring stress using imaging technologies and derived indices, and present current uses of machine learning to examine stress responses.

## 2. Phenotyping technologies to assess abiotic stresses

**Figure 2.**
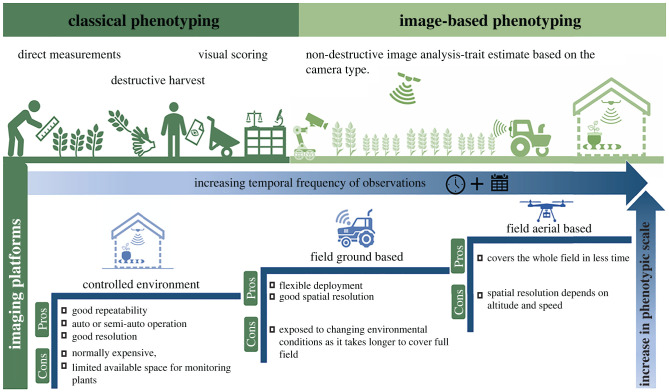
Schematic overview of phenotyping approaches and high-throughput phenotyping platforms across different environments and scales. Phenotyping approaches comprise classical and high-throughput methods. High-throughput imaging platforms span from those operating under controlled conditions to field-based conditions along with their advantages and limitations.

Phenotyping has a wide range of platforms spanning from those working under fully controlled conditions to field-based platforms (figure 2) or even platforms specifically designed to study specific traits (i.e. root traits, which are below ground). Numerous factors need to be considered when evaluating if a field-based or environmentally controlled-based platform is the most appropriate phenotypic system to study the underlying research question of the project, namely the scale of the intended work (genetic versus physiological study), the most adequate sensors deployed and their resolution, as well as associated costs and traits of interest (for further detail see the review from [18]).

Phenotyping the effects of abiotic stress in crops has conventionally been a relatively manual and laborious process. Classical methods for evaluating abiotic tolerance are based on destructive measurements. Destructive harvest includes splitting plants into segments, such as shoot from root, or by blade, sheath, stem and root. Destructive harvest allows measurement of traits such as shoot and root length, fresh and dry mass as well as yield components such as number of productive tillers or branches, fruit or grain weight etc [7,18]. Destructive phenotyping entails no highly specialized nor expensive equipment. However, it is usually labour-intensive, which, when shared with available space for growing plants, restricts the number of time points for sampling. Other classical methods include the use of hand-held equipment to evaluate plant performance under stress, including porometers to measure transpiration [19,20], infrared gas analysers to measure gas exchange [21] and soil and plant analyser development (SPAD) meters to determine the chlorophyll content and evaluate leaf damage under stress [22]. Transpiration and photosynthetic-related measurements should be performed at the same time of day to minimize circadian changes and executed on the same leaf and the same location on the leaves to reduce the effects of spatial variation, becoming arduous and time-consuming measurements. Another classical method to assess stress responses is the use of a visual scoring system. Visual scores evaluate the overall survival and/or vigor of the plant, are good indicators of the performance of the plant under stress and can be used to screen large populations. However, visual methods are not quantitative and are very subjective (different researchers may score the same plant differently). Phenotyping with destructive methods has become a major bottleneck in particular for studies dealing with large numbers of genotypes and sample sizes (e.g. forward genetics and breeding experiments). This phenotyping bottleneck has been unlocked by the advent of imaging techniques that have the potential to assess plant performance in a quantitative and time-series manner.

**Figure 3.**
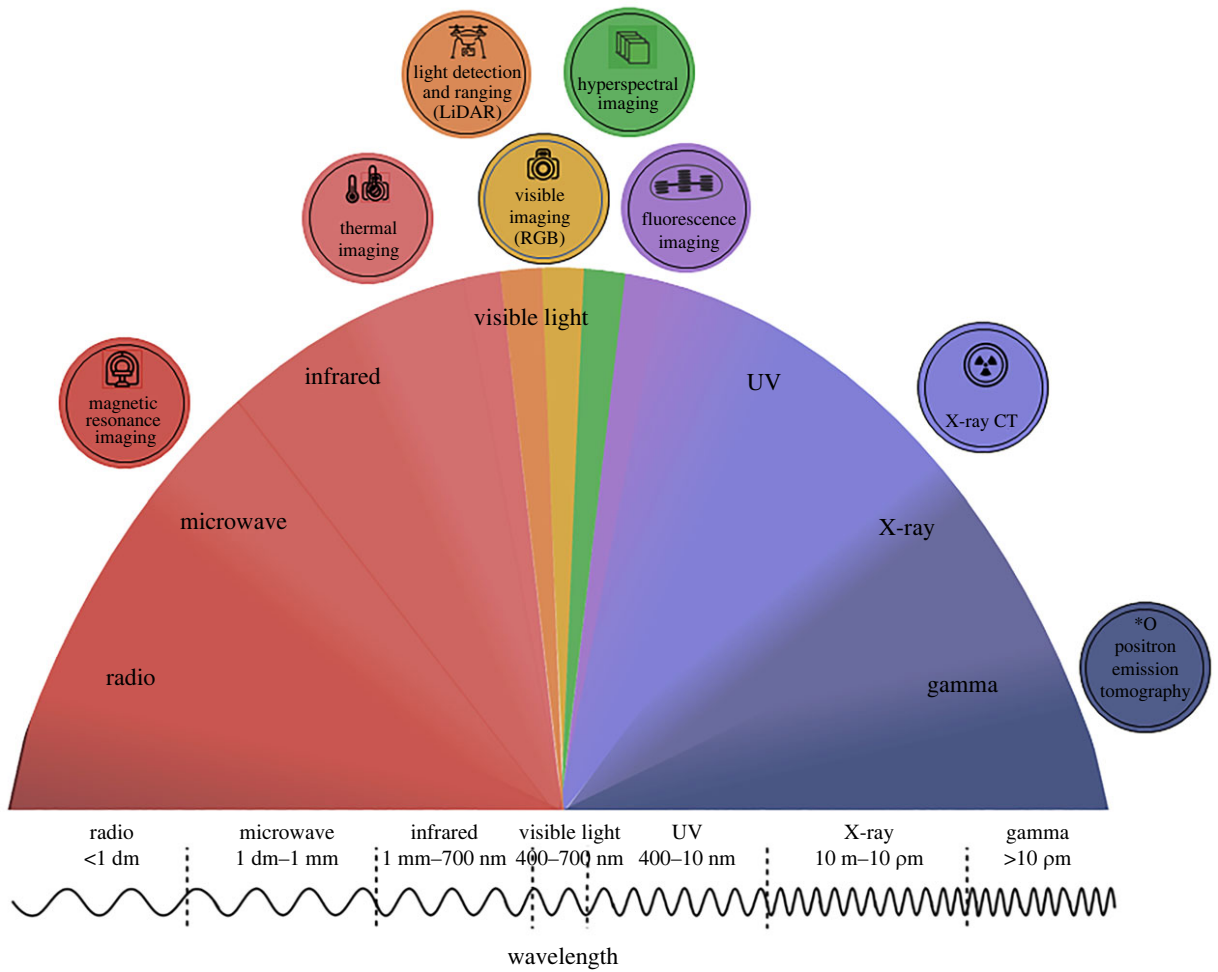
Phenotyping sensors across the electromagnetic spectrum showing wavelengths and frequencies. A variety of imaging technologies are available to capture signal from the visible and infrared spectrum of light. From left to right: nuclear magnetic resonance imaging (MRI) can acquire three-dimensional datasets of plant structures and be used in seeds and complete root systems growing in soil; thermal infrared (TIR) cameras are used for leaf temperature; light detection and ranging (LiDAR) (or laser scanner) is used to measure the three-dimensional distribution of plant canopies directly; visible imaging detects light in the visible range from ∼400 to 700 nm and is used to measure the morphological and colour properties of plants; hyperspectral imaging perceive hundreds of spectral bands with nm-level resolution between 350 and 2500 nm and are largely used in identifying plant stress; fluorescence imaging has been used as ultraviolet (UV) light in the range of 340–360 nm is reflected by different plant components as discrete wavelengths; X-ray computed tomography (X-ray CT) employs X-rays to produce tomographic images of specific areas of the scanned object; positron emission tomography (PET) is a nuclear imaging technique that produces a three-dimensional image or picture of a functional process. It can non-invasively image the distribution of labelled compounds, such as 11C 13 N or 52Fe.

Several imaging techniques and sensors have been used to precisely capture stress responses (table 1 and figure 3). The most straightforward and accessible of these are red, green and blue (RGB) sensors, cameras that capture the visible light spectrum (figure 3). Historically, visual phenotyping has been the key method for the artificial selection of crops into the food we know today. Now, paired with automated imaging and processing, RGB imaging remains the most versatile form of image phenotyping.

**Table 1.** Summary of available imaging sensors in plant phenotyping, including their advantages and related challenges.

sensor	traits measured	advantages	challenges	reviewed by
MRI	water status, transportation, and root architecture	three-dimensional architecture	low throughput and high cost	Pflugfelder *et al.* [23]
thermal	leaf/canopy temperature	temperature changes indicates water stress	highly influenced by environmental factors	Xie & Yang [24]
LIDAR	height and canopy architecture	high data resolution, can be operated at night	vast volumes of data, difficult analysis	Lin [25]
visible imaging (RGB)	root/shoot biomass, morphology, colour	low cost, monitoring of biomass, morphometry, and yield traits	unable to detect changes in water content or subtle	Li *et al*. [11]
hyperspectral imaging	traits vary depending on wavelength range of the sensor (examples include pigment concentration water content and plant nutrients); several spectral indices available (e.g. NDVI)	larger range of wavelengths, capturing stress signals before becoming visible	creates vast amounts of data; requires data mining and ML to improve data analysis	Liu *et al.* [26,27]
chlorophyll fluorescence	photosystem II activity	changes in ChF can occur before most other signs of stress	dark adapted measurements required	Maxwell & Johnson [28]
X-ray CT	root architecture	high-resolution, three-dimensional architecture	low automation and low throughput, high cost	Tracy *et al.* [29]
PET	translocation and transport of elements	shows movement and path of positron through the plant	low throughput, high cost	Garbout *et al.* [30]

While discussing image-based technologies for plant phenotyping, a threshold should be established to determine whether an experiment is either low-throughput or high-throughput both in terms of the number of phenotyped plants and overall cost. One may consider a low-throughput phenotyping experiment when a researcher is performing a physiological study (e.g. mutant or discriminant genotypes characterization) using a small number of plants (up to dozens). On the other hand, one may consider a high-throughput experiment when a researcher is performing a genetics study (e.g. quantitative trait loci-QTL- mapping, or genome-wide association studies- GWAS) or a breeding trial that involves several hundred to thousands of plants being phenotyped. The cost threshold can be defined based on the overall cost of the imaging device used to record the data for the experiment. One may consider a low-cost phenotyping experiment if the budget stands in the hundreds of US dollars. For example, a low-cost imaging sensor (i.e. Raspberry Pi) or a hand-held device such as a cellphone or a digital camera, yet low-cost equipment will produce low-throughput phenotyping due to the bottleneck caused by image acquisition. If the available budget ranges from hundreds to several thousand of US dollars, one may consider a medium cost phenotyping experiment, which is typically used in field trials with RGB and multispectral sensors mounted in unmanned aerial vehicles (UAV, i.e. ‘drones’), but where the researcher(s) can phenotype thousands of plants in a high-throughput manner. Image-based phenotyping can be considered of high cost if the available budget ranges from thousands to millions of US dollars, using expensive hardware to collect data on a much larger scale and without interruption in purpose-built facilities (e.g. imaging suites and field ground-based platforms). Finally, the running costs of a phenotyping platform should also be taken into consideration. A low-cost equipment can be easily handled by a researcher whereas purpose-built facilities require specialized staff and other running costs such as electricity or equipment maintenance.

RGB cameras can be used in a wide range of capacities, from automated germination assays [31], height measurements, biomass, morphology, flowering time and even plant identification aiding citizen science. RGB imaging remains the most accessible of sensors as the ever-improving quality of mobile phone cameras can be paired with open-sourced software such as ImageJ [32] or PlantCV [33] to provide low-throughput phenotyping at a very low cost. The use of low-cost equipment has been demonstrated by [34], where a series of RGB sensors were used to create a portable phenotyping platform to image maize shoots for 3D reconstruction. RGB imaging is also vital to more expensive phenotyping operations and its versatility is apparent in the range of data that can be collected. RGB data can provide estimates of biomass, germination and plant health of crops when used on a large scale or can be used for in-depth analysis on a small scale, monitoring growth morphology or even providing three-dimensional modelling [12,14,35]. In RGB imaging, pixels are captured and subsequently allow the measurement of shoot area and inferred mass, plant height and width, canopy density, other morphometric data, leaf colour and senescence. Therefore, enabling the quantification of differences in growth rates or senescence over a period of time, for example, under stress compared to control conditions [36–38]. In addition, RGB allows objective quantification of colour change and changes in leaf structure, which traditionally rely on human judgement and hence are prone to errors. For example, nitrogen deficiency, salinity, and water stress causes leaves' senescence, and RGB cameras allow the quantification of this stress symptom by counting the number of yellow pixels [39,40]. Also, leaf curving, which is a drought adaptation by reducing transpiration, has been quantified by imaging maize in the late afternoon (rolled leaves) and at pre-dawn the following day (unfolded leaves) and comparing the two images 45 days after planting [39].

Chlorophyll fluorescence (ChF) imaging sensors can provide false-coloured images of whole plant/leaf, enabling to estimate plant or canopy health in response to abiotic stress (e.g. [38,41–45]). Light absorbed by chlorophyll is used by plants in three ways: (i) photochemistry; (ii) non-photochemical quenching (i.e. heat dissipation); and (iii) re-emission as chlorophyll fluorescence. Thus, measuring the light emitted provides a proxy to determine the current photochemical efficiency of the plant. Chlorophyll fluorescence imaging provides key parameters such as the potential quantum yield of photosystem II (Fv/Fm), which is highly altered by both abiotic and biotic stress [46,47]. Steady-state chlorophyll fluorescence imaging has been applied for measuring plant photosynthetic capacity and its impact under salinity [48]. Other imaging technologies include infrared sensors that create false-colour thermal images that can identify stress before it can be identified in the visible spectrum as temperature provides a proxy measurement for stomatal conductance, which plays a major role in response to most stresses (as reviewed by [49]). Thermal infrared imaging (TIR) has been used to measure canopy temperature, providing an indirect calculation of stomatal conductance and transpiration of plants under salinity stress [50].

Image-based technologies also include three-dimensional measurements. Light detection and ranging (LiDAR) use laser scanners to create accurate and detailed three-dimensional models by measuring the distance between the sensor and a target. LiDAR has a wide scale application from small plants to forest stands, yet it can be costly and requires a longer imaging time hence longer battery life. Detailed three-dimensional LiDAR images combined with RGB images, chlorophyll fluorescence, photochemical reflectance index and leaf temperature images enable the evaluation of responses of pigments, photosynthesis, transpiration, stomatal opening and shape to environmental stresses, making this a great tool to exploit when monitoring plant responses to abiotic stress (as reviewed by [51,52]). Previous research used LiDAR to estimate stress impact (e.g. [53,54]), yet data collection and preprocessing are challenging and time-consuming.

Other three-dimensional image-based technologies include X-ray computed tomography (CT), positron emission tomography (PET) and magnetic resonance imaging (MRI) (as reviewed by [11,55–57]). These technologies are used at a plant level, providing quantitative high-resolution detection of structural damages induced by stress. X-ray CT imaging has been considered as the most suitable technology for *in vivo* structure phenotyping due to its relatively low cost and high spatial resolution and has been extensively used in root phenotyping (as reviewed by [55,56]). For example, X-ray CT scanning has been used to monitor the belowground development of potato tubers in response to combined heat and drought stress [58], or barley root growth responses to different N soil compositions [59]. Furthermore, X-ray CT imaging has also been used to evaluate seed morphology in wheat spikes in response to heat and drought [60]. PET is also a non-destructive imaging technology that can detect the location of small amounts of short-lived radioactive substances injected into an organism. Combining CT with PET provides valuable qualitative and quantitative information on soil structure and root growth at high resolution [30]. For example, Ruwanpathirana *et al*. [61] used PET and CT imaging to assess barley responses to low nutrient availability, monitoring in real-time the temporal dynamics of ^22^Na. MRI is based on radiowaves, allowing imaging of the protons of water (i.e. the internal physiological processes occurring *in vivo* [62]). For example, MRI has been used to assess relative differences in water distribution across the root tissues of cultivated barley (*H. vulgare*) and a halophytic barley (*H. marinum*) in response to salinity stress [63].

Hyperspectral imaging captures narrow wavelength bands within and beyond the visible spectrum, including visible and near-infrared (VNIR) and shortwave-infrared (SWIR), and can detect changes in reflectance associated with stress before any symptoms appear within the visible wavelength (as reviewed by [12,35,64,65]), whereas the SWIR data of Mertens *et al*. [66] can be used to measure a whole range of plant constituents, including nutrients, water or even secondary metabolites like flavonoids and terpenoids [67]. Mohd Asaari *et al*. [68] used an alternative data-driven method of hyperspectral image analysis to differentiate between water-stressed and control plants as early as 3 days after the start of the experiment. By being able to differentiate between stressed and control plants so early permits the length of the experiment to be reduced, allowing a larger number of experiments to be carried out and reducing the cost of individual experiments [68]. Further detail on stress traits, indices and applications will be discussed in §§5–6. Hyperspectral imaging usually requires advanced technical expertise in image segmentation and data analysis, for example, Behmann *et al*. [69] investigated the early onset drought responses of barley with SWIR imaging using a combination of both unsupervised and supervised machine learning (ML) methods. Moghimi *et al*. [70] used hyperspectral imaging and proposed the use of vector-wise similarity measurements and Bayesian inference to calculate posterior probability of salt class in pixels. The adoption of these methods enabled clear distinction of salt-treated from control plants after a very short time, in fact for one of the accessions as early as one day after salt imposition, facilitating the obtention of a quantitative ranking of wheat accession based on their salt tolerance. Thus, hyperspectral imaging is far less accessible than the previously mentioned imaging techniques due to the high cost of sensors and the vast amount of high-dimensional data produced, often requiring multidisciplinary teams and computer science to solve its big data challenge (see §7).

## 3. High-throughput phenotyping

Breeders heavily rely on phenotyping, using large number of genotypes and targeting the collective study of multiple traits simultaneously under a given condition. The challenge rests on breeding for yield and other traits, such as stress tolerance, using the most adequate phenotyping strategy for the trait of interest among a plethora of available phenotyping strategies (as reviewed by [18,71]).

High-throughput phenotyping (HTP) aims to characterize the full set of phenotypes by non-destructively, capturing plant traits and integrating biology with computers and robotics (as reviewed by [15,72,73]). High-throughput experiments can be established in both controlled and field environments by massively relying on frequent, non-invasive automated sampling and/or imaging of several hundred to thousands of plants in a short time frame [15,72,74,75]. HTP facilities with high cost can allow full control of the environment including temperature, light intensity and even day length, reducing spatial variation and optimizing their use to assess abiotic stress responses. These HTP platforms are automated and multifunctional, enabling the functional characterization of plants’ genomics (e.g. genomic selection and genome-wide association studies). Big data generated by HTP temporal platforms requires efficient data management, storage and analysis [76]. HTP, in general, uses imaging techniques to monitor plant growth and dynamic responses under stress in real time. The use of HTP has allowed the dissection of the genetic components of abiotic stress through time, which is not achievable via conventional phenotyping methods as discussed in §4.

Global investment in infrastructure for HTP facilitates the community progressing across the whole phenotyping pipeline. Many regional organizations have been established such as the Australian Plant Phenotyping Facility (APPF, https://www.plantphenomics.org.au/), EPPN (European Plant Phenotyping Network, http://www.plant-phenotyping-network.eu/), and NAPPN (The North American Plant Phenotyping Network, https://www.plantphenotyping.org) to name a few. Other initiatives to develop suitable synergies in phenotyping and efficiently access opportunities include the International Plant Phenotyping Network (IPPN, https://www.plant-phenotyping.org/) and EMPHASIS project (https://emphasis.plant-phenotyping.eu/).

HTP imaging techniques is still an emerging field in plant stress and has barely been tapped into to achieve their full potential. Other automated features of various HTP platforms are programmable watering to weight of plants to enable large-scale experiments requiring controlled watering levels. To our knowledge, few reports have evaluated transpiration and transpiration use efficiency (TUE) at the whole-plant level using mechanized gravimetrical methods [77,78], yet HTP has been successfully used to assess TUE in rice in response to salinity [37] and drought [79]. Moreover, open-source phenotyping technologies and off-shelf solutions are being established and becoming widespread, permitting easier practice and application for both researchers and farmers (e.g. [80–83]).

This review centres on the integration of HTP imaging and computing technologies to extract phenotypic traits for improving crop yield, quality and adaptability to abiotic stresses. We also focus on both controlled and field conditions. Field phenotyping platforms can be generally split into two categories the ground-based and the aerial-based platforms. Ground-based platforms can generate higher resolution data since they can capture images at a nearer range relative to the plants [84,85]. Aerial-based platforms can be quicker in capturing and measuring traits of a larger field plot. These two platforms have their advantages and limitations when used, which are summarized in figure 2.

There is still a serious need to develop suitable synergies in plant phenotyping to efficiently access opportunities, employ new technologies and determine data management arrangements that allow data exchange across installations, locations, and experiments. The implementation of new technologies needs to be integrated into the pipeline for all users in industry and academia alike.

## 4. Use of imaging to analyse abiotic stress

As previously mentioned, breeders have a hard task ahead of them to keep pace with higher yield under stressful conditions in response to a changing environment. Thus, all potential tools and technologies that may help overcome the seemingly insurmountable challenges of crop production in the future should be considered. This is the reason why many researchers are now using image-based technologies to improve the process of phenotyping. Image data paired with accurate high resolution environmental data provides information on the environment-genotype relationship in detail never before accessible [18]. Yet, the use of HTP in large-scale breeding programmes or to assist genomic selection is relatively new and full of challenges [71,86,87]. For example, Watanabe *et al*. [88] used RGB and near-infrared (NIR) cameras to study sorghum plant height in response to nitrogen availability, and was able to find a higher correlation between data collected with HTP and field measurements in the plots with lower fertilization, demonstrating that HTP measurements in field conditions are suitable for genomic prediction modelling. Rutkoski *et al*. [89] performed genomic selection to improve wheat grain yield using HTP to assess canopy temperature in five field environments with different irrigation regimes and sowing dates to expose wheat to heat stress.

Nevertheless, we consider that HTP offers tremendous potential in breeding experiments if one can combine rapid cycle genomic selection with imaging platforms to collect data under a multitude of environmental stresses. Hence, to help incoming researchers in reviewing image-based technologies that have been used by different authors to study abiotic stresses, we provide a comprehensive summary of imaging applications that targeted traits contributing to stress tolerance (table 2).

**Table 2.** Summary of research work that used imaging technologies to specifically study abiotic stress.

stress	growing conditions	species	year	imaging	traits measured	destructive (non-HTP) measurements	instrument/Phenotyping platform	reference
cold	controlled	pea	2015	RGB—fluorescence	biomass/growth related traits—chlorophyll fluorescence parameters	—	PlantScreen Photon Systems Instruments (PSI), Czech Republic	Humplík *et al.* [14]
cold	field	maize	2019	RGB—multispectral	stress detection, quantification and classification	—	Multispectral UAV, field of ICAR-NEH at Indian states of Meghalaya, India	Goswami *et al.* [90]
drought	controlled	barley	2014	RGB	biomass/growth related traits, plant hue	shoot biomass, tiller number, height	The Plant Accelerator, Adelaide, Australia	Honsdorf *et al.* [91]
drought	controlled	wheat	2015	RGB	biomass/growth related traits	—	The Plant Accelerator, Adelaide, Australia	Parent *et al.* [92]
drought	controlled	rice	2018	RGB	biomass/growth related traits, plant hue, architectural traits	shoot biomass and yield and yield components	High-throughput rice phenotyping facility at Huazhong Agricultural University, China	Guo *et al.* [93]
drought	controlled	green millet and foxtail millet	2015	RGB—fluorescence—near-infrared (NIR)	morphological traits—photosynthetic efficiency and chlorophyll fluorescence parameters—tissue water content	—	Bellwether Phenotyping Platform at the Donald Danforth Plant Science Center, USA	Fahlgren *et al.* [12,13]
drought	controlled	barley	2019	RGB	biomass/growth related traits, architectural traits	shoot biomass, plant height, tiller number	The Plant Accelerator, Adelaide, Australia	Pham *et al.* [94]
drought	controlled	barley	2019	RGB—fluorescence	height—chlorophyll fluorescence parameters	shoot biomass, relative water content	PlantScreen Photon Systems Instruments (PSI), Czech Republic	Marchetti *et al.* [43]
drought	controlled	rice	2020	RGB—near-infrared (NIR)—infrared—fluorescence	biomass/growth related traits, plant hue, architectural traits—water content—plant temperature—photosynthesis efficiency	—	LemnaTec, GmbH, Aachen, Germany	Kim *et al.* [79]
drought	controlled	lettuce	2020	RGB—fluorescence	biomass/growth related traits moprhological traits—chlorophyll Fluorescence parameters	—	PlantScreen Photon Systems Instruments (PSI), Czech Republic	Sorrentino *et al.* [44]
drought	controlled	maize	2018	hyperspectral	the leaf angle and surface area	—	PHENOVISION HTPPP located in the greenhouse of the VIB-UGent Center for Plant Systems Biology (Ghent, Belgium)	Mohd Asaari *et al.* [68]
drought	controlled	maize	2019	hyperspectral	vegetation indices	—	PHENOVISION, the HTPP infrastructure located at VIB, Ghent, Belgium	Asaari *et al.* [95]
drought	controlled	barley	2019	RGB	biomass/growth related traits	shoot biomass	IPK Gatersleben, Germany	Dhanagond *et al.* [96]
drought and nitrogen deficiency	controlled	sorghum	2015	RGB—near-infrared (NIR)	biomass/growth related traits, plant hue, architectural traits—senescence (%), NIR, water content composition parameters	shoot biomass, leaf area, plant height, dry matter content (%), moisture content (%), chlorophyll content	The Plant Accelerator, Adelaide, Australia	Neilson *et al.* [39]
drought and nitrogen deficiency	field	wheat	2019	RGB	biomass/growth related traits, plant hue	—	PhénoField, applied research institute ARVALIS, France	Beauchêne *et al.* [53]
drought and nitrogen deficiency	controlled	maize—soya bean	2017	hyperspectral	NDVI, leaf water content, concentrations of macronutrients	biomass, concentration of macronutrients	University of Nebraska-Lincoln	Pandey *et al.* [97]
heat	controlled	mung bean	2019	fluorescence	chlorophyll fluorescence parameters	—	Wals, Germany (Model not given)	Basu *et al.* [98]
nitrogen deficiency	controlled	sorghum	2017	RGB	biomass/growth related traits, plant hue	ionomic profiling	Bellwether Phenotyping Platform at the Donald Danforth Plant Science Center, USA	Veley *et al.* [99]
nitrogen deficiency	field	barley	2017	RGB—multispectral—thermal	plant hue—Crop Senescence Index (CSI), Photochemical Reflectance Index (PRI), various vegetation indices, Water Band Index (WBI)	yield and yield components	Arazuri Station of the Institute of Agrifood Technologies and Infrastructures of Navarra (INTIA), Spain	Kefauver *et al.* [100]
nitrogen deficiency	controlled	wheat	2020	RGB—hyperspectral	biomass/growth related traits, morphological and architectural traits—vegetation indices relating to chlorophyll levels	shoot biomass and yield and yield components; chlorophyll content	Agriculture Victoria's Plant Phenomics Victoria, Horsham (PPVH), Australia	Banerjee *et al.* [101]
nitrogen deficiency	field	maize	2020	hyperspectral	NDVI	—	LeafSpec, developed by the Purdue Phenotyping Lab group, USA	Ma *et al.* [102]
nitrogen deficiency	field	maize	2006	multispectral	leaf reflectance	—	multi-spectral charge-coupled device (CCD) camera s mounted on a mobile liquid nitrogen sprayer	Noh *et al.* [103]
nutrient deficiency	field	alfalfa	2019	RGB—multispectral	NDVI, leaf area index, ground coverage	biomass, yield, plant height	UAVs and sensors mounted on a phenomobile, USA	Cazenave *et al.* [104]
salinity	controlled	rice	2014	RGB—fluorescence	biomass—shoot senescence (%)	shoot biomass, leaf Na^+^ and K^+^ concentration	The Plant Accelerator, Adelaide, Australia	Hairmansis *et al.* [105]
salinity	controlled	rice	2015	RGB—fluorescence	biomass—chlorophyll fluorescence parameters	leaf Na^+^ and K^+^ concentration	The Plant Accelerator, Adelaide, Australia	Campbell [48]
salinity	controlled	rice	2016	RGB	biomass/growth related traits	shoot biomass	The Plant Accelerator, Adelaide, Australia	Al-Tamimi *et al.* [37]
salinity	controlled	chickpea	2017	RGB	biomass/growth related traits	shoot biomass, plant height, leaf Na^+^ and K^+^ concentrations, flowering time, leaf chlorosis and necrosis, yield and yield components	The Plant Accelerator, Adelaide, Australia	Atieno *et al.* [106]
salinity	controlled	barley	2017	RGB	growth curve registration (statistics paper)	—	The Plant Accelerator, Adelaide, Australia	Meng *et al.* [107]
salinity	controlled	wheat	2018	RGB	biomass/growth related traits	leaf Na^+^ and K^+^ concentrations	The Plant Accelerator, Adelaide, Australia	Asif *et al.* [108]
salinity	controlled	rice	2018	RGB—fluorescence	biomass/growth related traits	shoot biomass, gas exchange parameters (photosynthesis, stomatal conductance and transpiration), chlorophyll concentrations	The Plant Accelerator, Adelaide, Australia	Yichie *et al.* [109]
salinity	controlled	wheat	2018	hyperspectral	NDVI and EGI	shoot and root biomass	University of Minnesota, Minneapolis, MN, United States	Moghimi *et al.* [70]
salinity	field	tomato	2018	RGB	biomass/growth related traits, prediction of yield and yield components	shoot biomass and yield and yield components	UAVs. King Abdullah University for Science and Technology, Thuwal, Saudi Arabia.	Johansen *et al.* [110]
salinity	controlled	lettuce	2019	fluorescence	chlorophyll fluorescence parameters	shoot biomass	PlantScreen TRANSECT XZ SYSTEM	Adhikari *et al.* [111]
salinity	controlled	okra (*Abelmoschus esculentus* L.)	2019	hyperspectral	plant and leaf segmentation	biomass, SPAD, sodium concentration, photosynthetic rate and transpiration rate	—	Feng *et al.* [112]
salinity	controlled	wheat	2017	hyperspectral	stress detection, vegetation indices, leaf segmentation	shoot and root biomass	hyperspectral camera (PIKA II, Resonon, Inc, Bozeman, MT 59715, USA)	Moghimi *et al.* [113]

The major advantage of image-based technologies is that they are non-destructive, allowing multiple measurements to be taken per plant and the identification of time-specific loci that might be missed if phenotyped at a single time-point (i.e. at harvest). Thus, HTP facilities have been used to perform association studies and dissect the genetic architecture of abiotic stress responses; however, the number of loci identified using HTP platforms and longitudinal data is still limited, being applied to few major crops and targeting mainly drought and salinity. The underuse of imaging technologies in association studies can be attributed to the relatively small number of HTP platforms that can operate with the high number of plants required to perform genetic studies, as well as the recent development of imaging techniques and sophisticated data analysis that requires larger and multidisciplinary teams. The few studies that have carried out association studies in crops offer promising results, including the identification of multiple and yet undiscovered QTL in response to abiotic stress. These studies include dissecting the genetic architecture of drought in barley [91,94,96], maize [114], wheat [92], lettuce [115] and rice [93,116] as well as identifying new QTLs associated with salinity stress in wheat [108,117], barley [118], rice [37,48] and chickpea [119]. The power of HTP to detect temporal stress responses is clearly illustrated by the work of Campbell *et al*. [48], where salinity stress was examined in rice. With the use of a longitudinal genome-wide association model, the authors discovered a region on chromosome 1 that regulates the fluorescence shift, which is indicative of the longer term mechanism of ionic stress, and the early growth rate decline associated with salinity stress [48]. Dhanagond *et al*. [96] used RGB imaging to analyse biomass growth patterns during both stress and recovery phases of 100 diverse two-rowed spring barley in response to drought during pre-anthesis, which enabled identification of drought-adaptive QTLs containing genes involved in dehydration tolerance, namely dehydrins (*Dhn*4, *Dhn*7, *Dhn*8 and *Dhn*9) and aquaporins (e.g. *HvPIP*1;5, *HvPIP*2;7 and *HvTIP*2;1). The power of HTP to identify genetic regions underlying stress responses is also illustrated by the work of Guo *et al*. [93] as the authors investigated rice under drought stress and identified 470 association loci, 93% of which were co-localized with previously reported drought-related QTLs.

It is important to note that the above studies were performed in controlled conditions due to the ability to better control the environment and ease of phenotyping; however, many QTLs that have been found in controlled studies do not translate to field and breeding programmes [84]. Field trials are an enormous undertaking to phenotype as they are laborious to manage and can be very expensive (depending on location and labour cost), which limits how much data can be collected. Nevertheless, HTP in the field offers breeders and researchers large quantities of data that can be recorded annually. Thus, a proper field design and spatial corrections can further build our knowledge of the accessions/cultivars being examined under stress in ‘real’ conditions [18].

HTP in the field involves sensors that can be mounted on vehicles (e.g. [104,120], flown over using UAVs (e.g. [100,110]) or even incorporated into large structures creating field phenotyping platforms (e.g. [53,121]). Field phenotyping systems can support RGB, thermal, multi or hyperspectral sensors. UAVs are currently the most common imaging technology for field phenotyping, facilitating the recording of various traits. Under abiotic stress conditions UAVs have been used to record traits such as canopy height or yield mass of tomato plants under salinity [110], maize canopy temperature under drought stress [122].

There is a great deal of debate surrounding the most appropriate technology for field phenotyping. Arguments in favour of UAVs suggest that this platform is more flexible and less expensive to purchase and operate, enabling to sow and image different field sites, or easily transport the UAV across multiple locations, as well as a reduced imaging time. On the other hand, fixed phenotyping platforms are less dependent on weather conditions, for example, image can be collected with high winds, which is a limitation for UAVs. Also, if a field is located in a controlled airspace location, the image collection by a UAV will be further complicated by authorizations permits; hence, reinforcing the argument of phenotyping using fixed field platforms. To reduce the costs of field phenotyping platforms, custom-made solutions have been developed such as the ‘Phenocart’ that comprises RGB and TIR cameras, coupled with a high-precision Global Positioning System (GPS) mounted on a bicycle [123]. The Phenocart has been successfully used at a large-scale breeding field phenotyping experiment targeting wheat under drought and heat conditions [86]. Other custom-made field imaging solutions may be as simple as assembling the sensors on a tractor frame, enabling to phenotyping at a low-cost hundreds of plots [124]. Using this system, Pauli *et al*. [125] evaluated cotton agronomic and quality traits as well as its canopy temperature under well-watered and drought conditions, which enabled assessment of QTL patterns within and across years and between different irrigation regimes. Fixed field phenotyping facilities can offer great potential for abiotic stress, for example, PhenoField includes slidable covers that protects plots from rainfall and has a structure over the field that allows the camera to be moved across it to phenotype all the plots independently of weather conditions, facilitating the phenotyping of crop species under drought [53]. However, phenotyping in the field for other abiotic stresses such as waterlogging, cold or heat can become challenging and expensive. To overcome such challenges imaging technologies can be used to mimic and model field conditions in indoor facilities. For example, Marchetti *et al*. [43] studied drought in barley at a RH of 40%, which is closer to field real conditions of water deficit, and used a density rate of 1000 plants m^–2^ to mimic a canopy effect, showing that it is possible to discriminate between tolerant and sensitive barley genotypes by estimating a project canopy height of a population in response to drought.

However, imaging for abiotic stress using is just a piece of the puzzle due its complex genetic nature and visual perception. In the next sections, we discuss the use of indices to evaluate stress responses and provide some examples of the use of ML to speed up the process of feature extraction and stress identification.

## 5. Advances in image analysis of phenotypic data and the use of indices to estimate stress

**Figure 4.**
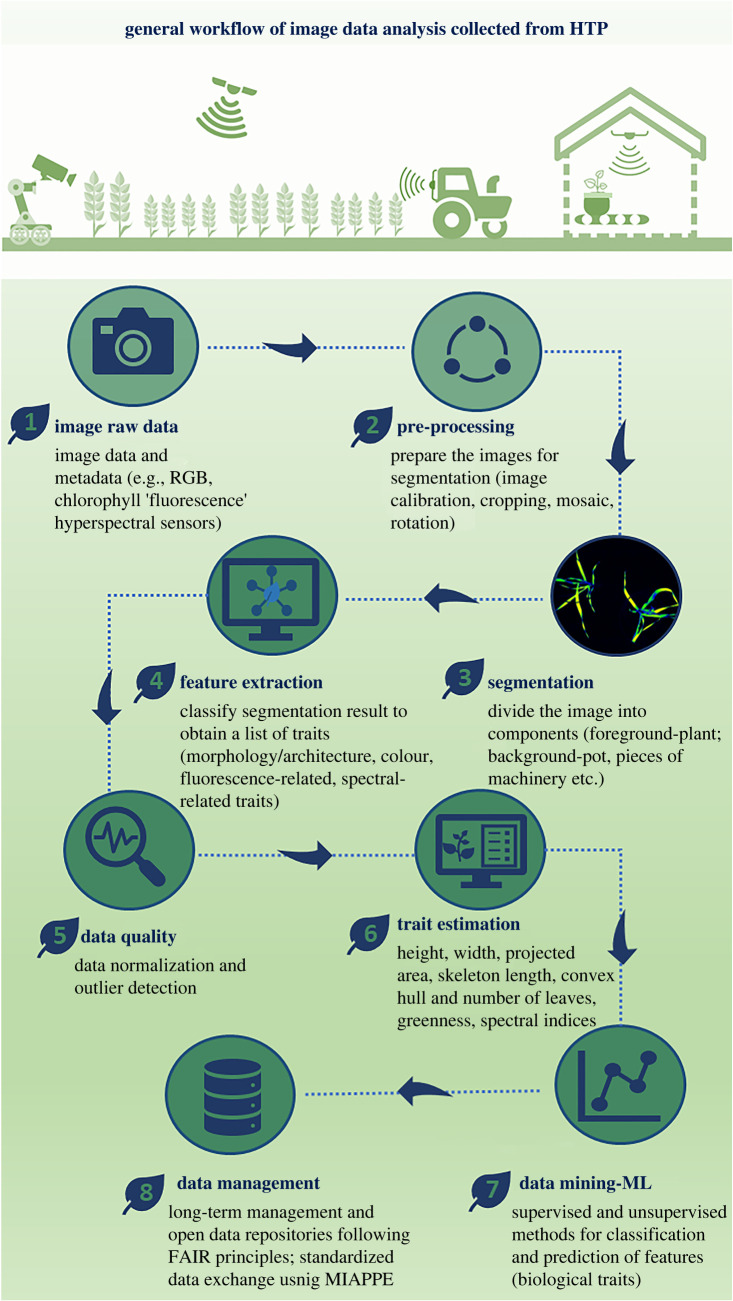
A general workflow for the high-throughput image data analysis. The workflow describes image data processing steps for the extraction of the quantitative traits. Summary of the workflow refers to steps: (1) image raw data; (2) pre-processing; (3) image segmentation; (4) feature extraction; (5) data quality; (6) trait estimation; (7) data mining; (8) data management.

Imaging has allowed many aspects of plant development, function and health to be monitored. A universal process of image analysis is difficult to achieve as imaging data formats may differ depending on the distinctive imaging sensors used (e.g. RGB, hyperspectral, multispectral, etc.). We summarize a workflow of image data analysis (figure 4) that includes: (1) Image capture in raw data format; (2) image pre-processing, contrast enhancement, noise removal, so that the image is manageable for the following step; (3) image segmentation to acquire the objects of interest (e.g. plant) and differentiate it from the background (e.g. pot and soil); (4) feature extraction to have the raw features according to the experimental targets, examples of this are gradient, edge, counter, shape, size, texture, corner point, colour features and so on; (5) data quality needs to be performed after extracting massive volumes of raw features; (6) traits selection and estimation is required to filter out and explore the important biological features; (7) data mining, for example, building dynamic growth using mechanistic models or the exploration of spatial and temporal information; (8) long-term management and integration of the vast amount of data and all essential metadata following the Findable, Accessible, Interoperable and Reusable (FAIR) principles [126].

Data acquisition information, protocols, environmental conditions, data description should be clear and easily accessible to support data discovery and data mining applications (further details can be found in [18]). By contrast to genomic data, phenotypic data are seldom reused. Having the means to reuse phenotypic data to perform large meta-analysis would grant an improved approach in dissecting the genetic architecture of traits across environments or breeding efforts. Several initiatives are creating tools for standardizing the description of phenotypic data. An example of one initiative to pave the way for data exchange and reuse is the ‘Minimal Information About Plant Phenotyping Experiment’ (MIAPPE, www.miappe.org) [127], which is a tool for common and clearly defined management standards and data formats.

During data trait selection and estimation, the plant image of each sample is converted into numerical data over the entire time series and prepared for analysis, meaning that deciding which statistical methods should be used to interpret the measurements can be complex. Therefore, traits selection and estimation of the image data need innovative and sensible analytical methods that can begin to interpret the responses of plants to abiotic stress from a structural, physiological and temporal sense. Structural traits can be estimated by computing the widely used colour-index based segmentation and classification, noise reduction and object composition. Such computed traits include, for example, the projected shoot area (PSA), which can be extracted from RGB images. The sum of PSA from all angles of images can be used to estimate shoot biomass as it is highly correlated with leaf area (LA), plant shoot fresh weight (FW) and dry weight (DW) [128].

Plant-image based technologies are non-destructive, allowing to image the same plant daily and record temporal or longitudinal data. Thus, it is possible to estimate plant growth with exponential growth models being typically used for young seedlings and short growth intervals. Yet, modelling of plant growth is extremely complex, and it is necessary to develop statistical models that can interpret and estimate HTP-derived traits with researchers exploring different modelling strategies. Numerous mathematical models have been used to describe growth curves of high-throughput data (e.g. [92,129]). For example, Campbell *et al*. [48] used a decreasing logistic curve as a proxy for plant growth estimation and identified several loci associated with salinity tolerance in rice by collecting daily RGB images. However, most of these models make assumptions about the shape of the curve. To improve growth modelling, different methods have been tested. Feng *et al*. [112] used a deep learning approach to improve image segmentation from hyperspectral images and predict growth of okra plants in response to salinity. Other authors have used spline functions to provide an unbiased analysis of HTP data as they have the advantage of making no *a priori* assumptions about the shape of the growth curve, becoming a more suitable method to estimate growth in stressed plants [37,130].

In the past years, the most popular method to extract crop information from digital imaging tools is the derivation of spectral indices, which enables estimation of physiological traits. The most used indices are vegetation indices (VI) also known as spectral vegetation indices (SVI). These indices are designed to denote relative density, crop health, moisture and nutrient content. VIs are essentially simple ratios between specific wavelengths, which are used to quantify various plant traits. The basis for VIs is to use the distinctive spectral signature of green vegetation as compared to spectral signatures of other objects such as soil, sand, exposed rock, concrete or asphalt that would commonly display a stable increase in reflectance (with no irregular jumps) as wavelength increases from the visible to the NIR. Green leaves and vegetation have a unique spectral reflectance pattern in the visible and near-infrared wavelengths. This makes it possible to distinguish between soil and vegetation, vegetation's vigor and vegetation properties (canopy biomass, absorbed radiation, chlorophyll content, etc.) [131,132]. Hyperspectral data analyses have provided more information from remotely sensed imagery than ever feasible before. Numerous VIs (table 2) have been proposed and are widely used by the phenotyping community due to their simplicity (usually simple algebraic formulations) and broad application [133,134]. Index DataBase (DBI) (https://www.indexdatabase.de/) is a tool for working with remote sensing indices and provides a quick overview of over 500 indices that are usable for a specific sensor and a specific topic, including stress assessment.

Among the VI indices, the normalized difference vegetation index (NDVI) [135] and simple ratio (SR), i.e. the ratio vegetation index (RVI) [136], are the most commonly used (table 3). SR is a ratio between the reflectance measured in the NIR and red bands, and it is used as a quick approach to characterize green leaves from other background objects in the image and estimate the relative biomass. If both the red and NIR bands have equal or comparable reflectance, then the SR is 1 or close to 1. SR values for soils are usually near 1, with the SR tending to increase as the detection of green vegetation increases in the image. It is important to note that SR values are not constrained, and their values can increase considerably beyond 1. NDVI is also calculated from reflectance measurements in the red and NIR portion of the spectrum, ranging from −1.0 to 1.0, where positive values indicate increasing greenness and negative values indicate non-vegetated features such as water, rocks, clouds, etc. Healthy vegetation should be above 0.5 and stressed plants are usually bellow this value. The NDVI has been used for decades in both field and greenhouses scenarios, yet it is not ideal for plants grown in artificial light conditions as this contains NIR to optimize photosynthesis. In such cases, the use of a single-image NDVI (SI-NDVI) to detect stress symptoms is advised [151]. Green normalized difference vegetation index (GNDVI) is calculated from reflectance measurements in green and NIR portion of the spectrum, ranging from 0 to 1.0. This index is related to the proportion of photosynthetically absorbed radiation and is linearly correlated with Leaf Area Index (LAI) and biomass [152]. Genetic variation for VIs was described in several studies [110,153–156]. VIs are used to associate important traits of cereal crops, such as grain yield under stressed conditions (e.g. [157–159]). Also, we should note that different environments have their own individual variable and properties, which should be taken into consideration when using different VIs. Selecting for specific VIs should be done with comprehensive thought and weighing in the benefits and limitations of established VIs when applying them in a combination that fits a particular environment. This allows for VIs to be tailor-made for specific experimental designs, platforms and stress applications. For example, Liu *et al*. [26,27] tested different hyperspectral sensors and used partial least square regression to carry out proximal sensing of N content in wheat, suggesting that the indices obtained by the authors can be further used to validate N quantification in other crops. For a more in-depth understanding of VI refer to reviews by Lowe *et al*. [64] and Xue & Su [160].

**Table 3.** Summary of the most used spectral indices for monitoring of crop stress. *ρ*RED, *ρ*GREEN and *ρ*BLUE, represent the spectral reflectance of red band, green band and blue band respectively. *ρ*NIR: reflectance of the near-infrared band. *ρ*SWIR: reflectance of the shortwave-infrared band. *ρ*MIDIR: reflectance of the mid-infrared band.

name	abbreviation	formula	description with related traits and challenges	references
difference vegetation index	DVI	NIR – Red	sensitive to the amount of vegetation; simplest ratio; does not deal with the difference between reflectance and radiance caused by the atmosphere or shadows	Jordan [136]
simple ratio	SR	*ρ*NIR/*ρ*RED	ratio of NIR scattering to chlorophyll and light absorption used for simple vegetation distinction	Jordan [136]
modified simple ratio	MSR	(*ρ*800 − *ρ*445)/(*ρ*680−*ρ*445)	a combination of renormalized NDVI and SR to improve sensitivity to vegetation characteristics	Chen [137]
modified red-edge simple ratio index	MRESR	(*ρ*750 – *ρ*445)/(*ρ*705 nm – *ρ*445)	vegetation for low nitrogen stress	Datt [138]
normalized difference vegetation index	NDVI	(*ρ*NIR – *ρ*RED)/(*ρ*NIR + *ρ*RED)	measuring green vegetation through normalized ration ranging from −1 to 1	Rouse *et al.* [135]
green normalized difference vegetation index	GNDVI	(*ρ*NIR − *ρ*GREEN)/(*ρ*NIR + *ρ*GREEN)	modification of NDVI, more sensitive to chlorophyll content	Agapiou *et al.* [139]
red-edge normalized difference vegetation index	RENDVI	(*ρ*NIR − *ρ*RedEdge)/(*ρ*NIR + *ρ*RedEdge)	modification to NDVI, using red-edge information to probe for changes in vegetation health	Gitelson & Merzlyak [140]
green optimized soil adjusted vegetation index	GOSAVI	(*ρ*NIR − ρGREEN)/(*ρ*NIR + *ρ*GREEN + 0.16)	variation of NDVI to reduce the soil effect	Sripada *et al.* [141]
optimized soil adjusted vegetation index	OSAVI	(*ρ*NIR − ρRED)/(*ρ*NIR + *ρ*RED + 0.16)	provides greater soil variation than SAVI for low vegetation cover	Sripada *et al.* [141]
green ratio vegetation index	GRVI	*ρ*NIR/*ρ*GREEN	related with leaf production and stress	Sripada *et al.* [141]
red, green ratio index	RGRI	*ρ*RED/*ρ*GREEN	relative expression of leaf redness caused by anthocyanin to that of chlorophyll	Gamon & Surfus [142]
nonlinear index	NLI	(*ρ*NIR2 − *ρ*RED)/(*ρ*NIR2 + *ρ*RED)	modification of NDVI used to emphasize linear relations with vegetation parameters	Goel & Qin [143]
leaf water content index	LWCI	log(1 − (*ρ*NIR − *ρ*MIDIR))/ −log(1 − (*ρ*NIR − *ρ*MIDIR))	moisture content of the leaf canopy	Ceccato *et al.* [144]
enhanced vegetation index	EVI	2.5[(*ρ*NIR – *ρ*RED)/(*ρ*NIR + 6 * *ρ*RED – 7.5 * *ρ*BLUE + 1)]	optimize the vegetation signal with improved sensitivity in high biomass regions	Huete *et al*. [145]
photochemical reflectance index	PRI	(*ρ*531 − ρ570)/(*ρ*531 + *ρ*570)	indicator of leaf and plant canopy photosynthetic efficiency	Gamon *et al.* [146]
structure insensitive pigment index	SIPI	(*ρ*800 − ρ445)/(*ρ*800 + *ρ*680)	Indicator of increased canopy stress (carotenoid pigment)	Pen̄Uelas *et al.* [147]
modified red edge NDVI	mRENDVI	(*ρ*750 − ρ705)/(*ρ*750 + *ρ*705 −2 * *ρ*445)	capitalizes on the sensitivity of the vegetation red-edge to small changes in canopy foliage content, gap fraction and senescence	Sims & Gamon [35]
normalized difference water index	NDWI	(*ρ*NIR − *ρ*SWIR)/(*ρ*NIR + *ρ*SWIR)	measures the change in the water content of leaves by using the NIR and SWIR bands	Gao [148]
moisture stress index	MSI	(*ρ*1599)/(*ρ*819)	sensitive to increasing leaf water content; used in canopy stress analysis and productivity prediction	Behmann *et al.* [69]
normalized difference infrared index	NDII	(*ρ*819 − *ρ*1649)/(*ρ*819 + *ρ*1649)	sensitive to changes in water content of plant canopies; used in crop agricultural management, forest canopy monitoring, and vegetation stress detection	Hardisky *et al.* [149]
plant senescence reflectance index	PSRI	(*ρ*680 − ρ500)/*ρ*750	an increase in PSRI indicates increased canopy stress (carotenoid pigment), the onset of canopy senescence, and plant fruit ripening; vegetation health monitoring, plant physiological stress detection, and crop production and yield analysis	Merzlyak *et al.* [150]

The stress tolerance of a plant can be estimated using various indices, which are calculated for any estimated trait (e.g. yield, PSA). Stress tolerance indices have been used for many crops [37,161–163] and new indices have been suggested (e.g. [164]). A more detailed explanation of different stress indices can be found in review papers such as Morton *et al*. [165]. Pour-Aboughadareh *et al*. [166] developed a user-friendly software- iPASTIC- to facilitate the different stress indices calculations when using large datasets. New VIs and stress indices will continue to be developed, which will greatly broaden abiotic stress research areas. Despite the simplification that VIs and stress indices can provide, the main challenge in phenotyping, in particular image-based phenotyping, remains on handling large volumes of data. This big data challenge has been approached by computer scientists as we will discuss next.

## 6. Machine learning approaches to analyse abiotic stress

As previously mentioned, imaging sensors have greatly evolved due to considerable technological advancements and their cost has significantly been reduced in the last decade. With rising quantities of data, researchers need to identify strategies for data management and storage. The wealth of information arising from imaging sensors requires specialized methods for processing, analysis and knowledge acquisition. To overcome the challenges accompanying data management, machine learning (ML) has become the leading method to accelerate data integration and detect stress phenotyping traits (see review by Singh *et al*. [167]).

ML can be defined as the ability of a computer program to imitate human-based learning without being explicitly programmed to do so [168]. This learning process is split into two categories: supervised and unsupervised learning. Supervised learning uses labelled data to train and test a model of an analyst's choice (e.g. stress versus control). By contrast, unsupervised learning does not require a training dataset, instead it uses unlabelled data and employs clustering techniques to analyse the features (i.e. the biological traits) of that dataset. ML has been extensively used in phenotyping, particularly in the detection, classification, quantification and prediction of plant diseases (see review by [169]). By contrast, the use of ML in abiotic stress is still underexplored, which is largely due to the complex mechanisms of abiotic responses that result in a less straightforward phenotype compared to a disease phenotype. For example, disease detection, classification and quantification are simplified due to the obvious lesion symptoms. In abiotic conditions such as drought or nitrogen deficiency, plants' responses are more subtle (e.g. leaf chlorosis), which means that a typical ML pipeline will struggle to interpret this subtle difference between a control and stressed plant.

ML algorithms work only on numerically based datasets and require computer vision to analyse images produced from a sensor. RGB sensors are arguably the most common type of imaging method due to their low-cost and flexibility of use. However, under abiotic stress conditions, the use of RGB images for ML analysis is limited due to the indistinguishable nature of symptoms in the visible spectra. On the other hand, spectral-based imaging sensors (hyperspectral or multispectral) are advantageous despite their complexity. ML in spectral data is more powerful than RGB data as stress signatures become more obvious in abiotic stress beyond the visible spectra (e.g. NDVI). The use of ML for spectral data analysis requires further steps of pre-processing the data so that ML algorithms can efficiently analyse each spectral image. Also, ground-truth data such as harvesting data, weather data or chlorophyll content are important to validate ML algorithms.

Depending on the experiment and the specific type of imaging sensor applied, various ML algorithms can be used. For example, if a researcher wants to classify images in terms of stress versus control, they can use different classification algorithms such as support vector machine (SVM) or random forest. This classification strategy has been used in hyperspectral imaging, enabling to estimate biomass changes in response to drought stress in barley (e.g. [69,170]). If a researcher wants to anticipate plants behaviour in response to an abiotic stress (i.e. ML prediction), algorithms such as artificial neural network have proven to be efficient in lettuce under drought conditions (e.g. [171]).

Even though ML is a powerful tool, the performance of ML algorithms begins to degrade when datasets become too complex. In HTP, when using hyperspectral sensors, there is a considerable higher number of traits along with the involvement of time series, resulting in an increase in data complexity. In such cases, the technical prowess of deep learning (DL) algorithms has become more appealing. DL can be defined as a class of ML algorithms whose structure composes multiple layers, which may be used to extract high-level features (traits) from a dataset. The structure of each DL model is inspired by the function of the human brain, mimicking the process of neurons sending and receiving signals to perform specific actions. DL harnesses a great potential for abiotic stress analysis (see review by [172]), yet due to the intricacy of its implementation and high data requirements, research using DL is still in its infancy in HTP for abiotic stress. Ghosal *et al*. [173] used a convolutional neural network (CNN) to analyse 25 000 RGB images of soya bean leaves subjected to several diseases and nutrient deficiency. This DL method enabled the identification and quantification of foliar stresses in a fast way that can be further applied in real-time stress detection consistently and accurately. Kaneda *et al*. [174] performed ML prediction using both environmental data and RGB images of tomato plants exposed to drought stress. The authors model prediction for drought stress was achieved using a combination of sliding window-based support vector regression and CNN [174]. In field conditions, multispectral data from 382 soya bean genotypes was collected with a UAV sensor and used to train a feed-forward neural network [175]. The results showed that this DL methodology was able to classify plants responses to flooding stress using a scoring of flooding visual symptoms [175].

Although ML models can produce an adequate performance on a small dataset, DL models cannot produce sufficient performance when trained on a small dataset as their architecture is too complex. This leads to an issue known as ‘Overfitting’, where a DL model will produce impressive results on a training dataset but will fail to generalize to un-seen data. The use of ML also requires the creation of labelled data for supervised learning as ML and DL models cannot generate their training/test datasets without appropriate annotations. The major challenge in ML and DL research in abiotic stress phenotyping is the lack of openly available data repositories. The lack of open-source data repositories limits the improvement of ML and DL algorithms by the larger scientific community because only research groups who have access to affordable imaging sensors can produce data of considerable size. Initiatives where large datasets are made available to the larger community are paving the way for faster development and improvement of ML and DL algorithms. Such open-source repositories with large datasets are transforming stress classification and prediction with the leading examples of the initiatives of Plant Pathology 2020 [176] and the platform for big data in agriculture (e.g. PlantVillage- https://bigdata.cgiar.org/divi_overlay/plantvillage-nuru/, verified 24th November 2021). A comprehensive list of available imaging datasets has been complied by [177]. However, many open-source repositories contain only disease data or a ‘low’ number of images from a ML and DL perspective, limiting abiotic stress research.

## 7. Conclusion and future perspectives

Imaging technologies are certain to be a great tool for large genetic studies (including breeding and association analysis), but for HTP to be more widespread, it is important to create additional imaging facilities worldwide as well as training a new generation of plant researchers that combine expertise in plant physiology and data analysis in collaboration with statisticians and computer scientists. Moreover, as the bottleneck shifts from phenotyping to data analysis there is an increasing need to develop accessible data analysis tools that are both accessible and easy to use by plant scientists and breeders. One may anticipate that the development of a new platform automatically collecting and registering multi-source data for phenotyping, and employing a user-friendly graphical user interface (GUI) will greatly benefit research and breeding communities.

## Data Availability

This article has no additional data.
